# Transcriptome Co-expression Network Analysis Identifies Key Genes Regulating Conchosporangia Maturation of *Pyropia haitanensis*

**DOI:** 10.3389/fgene.2021.680120

**Published:** 2021-06-30

**Authors:** Yinghui Lin, Kai Xu, Yan Xu, Dehua Ji, Changsheng Chen, Wenlei Wang, Chaotian Xie

**Affiliations:** ^1^Fisheries College, Jimei University, Xiamen, China; ^2^Fujian Engineering Research Center of Aquatic Breeding and Healthy Aquaculture, Xiamen, China; ^3^Key Laboratory of Healthy Mariculture for the East China Sea, Ministry of Agriculture, Xiamen, China

**Keywords:** *Pyropia haitanensis*, conchosporangia, weighted gene co-expression network analysis, diacylglycerol kinase, lipid metabolism

## Abstract

Conchosporangia maturation is crucial for the yield of *Pyropia/Porphyra*. However, the molecular mechanisms underlying this process are poorly understood. In this study, we selected two strains of *Pyropia haitanensis* that show significant differences in conchosporangia maturation as materials to produce RNA-Seq libraries. Then, we identified key molecular pathways and genes involved in conchosporangia maturation by conducting a weighted gene co-expression network analysis. Two specific modules were identified, and included functions such as phosphorus metabolism, lipid metabolism, and the phosphatidylinositol signaling system. The hub genes that responded positively during conchosporangia maturation encoded diacylglycerol kinase (DGK) and phosphatidylinositol-3-phosphate-5-kinase, which are involved in the synthesis of phosphatidic acid, a key component of lipid metabolism. A full-length *DGK* sequence of *P. haitanensis*, designated as *PhDGK1*, was obtained by rapid-amplification of cDNA ends. Conserved motif and phylogenetic tree analyses showed that *PhDGK1* belongs to DGK Cluster II. The transcript level of *PhDGK1* increased during conchosporangia maturation in both strains, but increased earlier, and to higher levels, in the early-maturing strain than in the late-maturing strain. This pattern of gene expression was consistent with the patterns of maturity and changes in pigment contents. These results indicate that lipid metabolism plays a key role in regulating conchosporangia maturation in *Pyropia* spp., and that *PhDGK1* might be a useful molecular marker for breeding new early-maturing strains.

## Introduction

*Pyropia*/*Porphyra* contains substantial amounts of free alanine, glutamic acid, aspartic acid, and glycine, and is a popular foodstuff source for the locals in certain coastal areas of China, Japan, and Korea. *Pyropia haitanensis*, one of the most commercially important *Pyropia*/*Porphyra* species, is widely cultivated along the coast of South China (Xie et al., 2008). According to the Fisheries and Administration Agency of China, *P. haitanensis* production in 2019 reached 150,000 tons dry weight, accounting for 75% of total *Pyropia* production in China (Fisheries and Administration Agency China, 2020). The output of *P. haitanensis* in China is increasing, and this aquacultured crop has important economic and ecological value (FAO, 2017). The complete life history of *Pyropia* consists of the microscopic conchocelis stage (sporophyte) and the macroscopic thallus stage (gametophyte) (Xu et al., 2015). The thallus releases carpospores that develop into the conchocelis, and then conchocelis developed into conchosporangia. The mature conchosporangia release conchospores under suitable conditions. The conchospores undergo meiosis during the first or first two cell divisions and develop into thalli (Blouin et al., 2011). Therefore, the maturation of conchosporangia strongly affects the amount of conchospores, which then affects the yield and quality of *P. haitanensis* thalli. Whether the conchosporangia mature readily is a key factor in the breeding of new varieties.

Additionally, it is critical to manipulate the culture conditions to control the growth and reproduction of the conchosporangia stage, and this plays a major role in the industrial seeding of *Pyropia.* To obtain a steady culture of conchocelis and induce healthy conchospores, light intensity, temperature, and the phosphorus concentration are fundamental factors, both in laboratory-scale and industrial-scale cultivation (Sidirelli-Wolff, 1992). The formation of conchosporangia can be promoted by shortening the photoperiod and decreasing light intensity. The suitable light level for conchosporangia maturation is between 1,000 and 1,500 lux, with a light cycle of about 10 h (Green and Neefus, 2014). Changes in the ambient temperature also affect the formation of conchosporangia. Previous studies have shown that there is a correlation between the number of conchosporangia formed and temperature between 26 and 30°C, with 28°C being the optimum ripening temperature for *P. haitanensis* (Xie et al., 2013). Notably, the number of conchosporangia was found to increase when the ratio of nitrogen to phosphorus in medium was about 2–1 and the phosphorus concentration was between 2 and 120 μM/L (Frazer and Brown, 1995; Chen et al., 2005). However, the mechanisms regulating conchosporangia maturation are still unknown.

Lipids, one of the important bioenergy storage materials providing an energy stock for metabolism, are the major constituents of biological membranes. In plants and seaweeds, membranes can sense extracellular conditions and initiate signaling in response to various environmental stresses (Okazaki and Saito, 2014). After the membranes sense changes in the external environment, activation of lipid transduction enzymes initiates phospholipid signal transduction. Phosphatidic acid (PA), a lipid signaling molecule, accumulates to positively regulate signal transduction after sensing environmental changes (Arisz et al., 2009). Phosphatidic acid is formed via activation of the phospholipase C (PLC)/diacylglycerol kinase (DGK) pathway, and PA accumulates rapidly when the abundance of DGK increases. In plants, several PA-binding proteins have been identified, such as the protein kinase PDK1. In *Arabidopsis*, PA regulates root hair development. The external addition of PA was found to alter the activity of AGC kinases in an *AtPDK1*-dependent manner, indicating that phospholipid signaling is upstream of *AtPDK1 in vivo* (Anthony et al., 2004). Wada et al. (2020) detected higher phosphatidylinositol (PI) content in pollen of a heat-tolerant cultivar than in pollen of a heat-sensitive cultivar under high temperature conditions, suggesting that PI is a precursor of phosphoinositide, which activates rice pollen germination and tube growth under high-temperature conditions. A study on the seaweed *Sargassum horneri* showed that its lipid composition changes during growth. The total contents of lipids, including monogalactosyldiacylglycerols, digalactosyldiacylglycerol, lyso-SQDG, and 15 1,2-diacylglyceryl-*O*-2’-(*N*, *N*, *N*-trimethyl)-β-alanines, were found to initially increase and then decrease during growth of *S. horneri* (Zhang et al., 2017). Therefore, lipids are crucial for the growth and development of higher plants and seaweeds.

Lipids are also involved in the response to abiotic stresses in *P. haitanensis*. Wang et al. (2015) found that the levels of phosphatidylcholine and lysophosphatidylcholine increased during *P. haitanensis* conchosporangia formation, while the levels of sphinganine decreased. The fatty acids C18:2, C20:4, and C20:5 in *P. haitanensis* are oxidized to oxylipins, and further produce short-chain volatile components that function as either defense compounds or secondary signal molecules in algal defense (Chen et al., 2018). 1-Octen-3-ol, as a self-stimulating oxylipin messenger, enhances the synthesis of methyl jasmonate (MeJA), indole-3-acetic acid (IAA), and gibberellin A3 (GA_3_) to adjust the cellular redox state and promote primary metabolism and cell growth (Chen et al., 2019). However, the role of lipids in the regulation of filament maturation in *Pyropia* spp. is unclear.

In the present study, we selected two strains of *P. haitanensis* that show significant differences in conchosporangia maturation. The photosynthetic pigment content and ratio of conchosporangia at certain time points were determined for both strains, and these data were used to select the appropriate time points to collect samples for high-throughput sequencing. Further analyses were carried out to gain insight into key molecular pathways and genes using weighted gene co-expression network analysis (WGCNA). The functions of key genes were then analyzed via gene cloning and differential expression analysis. These results provide new information about the mechanisms of conchosporangia maturation in *Pyropia* spp.

## Materials and Methods

### Plant Materials and Culture Conditions

The S-1 strain and S-2 strain of *P. haitanensis*, purified progeny of doubled haploid (DH) lines, were selected and purified by the Laboratory of Germplasm Improvements and Applications of *Pyropia* at Jimei University, Fujian, China. The conchocelis without conchosporangia were cultured at 29°C under 20 μmol photons m^–2^ s^–1^, 9L:15D, and 30‰ salinity to induce conchosporangia maturation. Each treatment consisted of triplicate cultures with an initial quantity of 0.05 g (fresh weight) conchocelis without conchosporangia. The cultures were performed in 250 mL sterilized filtered seawater enriched with 1.36 mg L^–1^ P (NaH_2_PO_4_) and 14 mg L^–1^ N (NaNO_3_), with the medium renewed every 7 days. The percentage of conchosporangia to total conchocelis was determined according to Xu et al. (2017). The conchocelis area (mm^2^) and conchosporangia density (number of conchosporangia colonies per mm^2^ of conchocelis area) were assessed using a microscope (Nikon Eclipse 80i, Japan) every 7 days when the medium was renewed (Xu et al., 2017).

### Determination of Pigment Content

We used the methods of Zhong et al. (2016) to quantify the phycobiliproteins phycoerythrin (PE), phycocyanin (PC), and allophycocyanin (APC).

### RNA Extraction, Transcriptome Sequencing and Assembly

Total RNA was extracted from the conchocelis using the Plant RNA Kit R6827 (Omega, Germany) according to the manufacturer’s instructions. The RNA concentration and RNA quality were assessed using NanoPhotometer and NanoDrop instruments, respectively. For each sample, 2 μg RNA was used as input material. A total of 27 RNA-Seq libraries were generated and then sequenced on an Illumina HiSeq2000 instrument (Guangzhou Gene Denovo Biotechnology Co., Ltd., China).

The raw reads were filtered by removing reads with adapters, reads with a ratio of N (percentage of nucleotides in the reads that could not be sequenced) >10%, and low-quality reads (those containing over 40% bases with *Q* value < 20%). Reads that mapped to ribosome RNA were also removed. The longest transcript of each gene was selected as the unigene. Filtered reads from all 27 samples were assembled using Trinity software (Grabherr et al., 2011). Gene transcript levels were determined as the number of uniquely mapped reads per kilobase of transcript per million mapped reads (RPKMs).

### WGCNA Analysis and Network Construction

For the WGCNA, unigenes obtained from the RNA-seq data were filtered for a second time. Genes with RPKM < 1 in all samples or with a coefficient of variation [(SD/Mean) × 100%] <0.1 were removed. All the related unigenes were used for WGCNA with the R-package (Langfelder and Horvath, 2008). According to the correlations between genes, the co-expression adjacency matrix was formed and converted into a topological overlap matrix (TOM). Co-expression modules were generated by hierarchical clustering and a dynamic tree cut. The minimum module size was set to 50 and modules with a tree height <0.3 were merged together. The expression patterns of 12 modules were displayed as the eigenvalues (equivalent to the weighted synthesis values of all genes in each module, which reflects the comprehensive expression level for the module). The turquoise module-based networks for lipid metabolism and PI signaling system were constructed using genes annotated in these pathways as nodes to extract the co-expressed gene pairs. The resulting networks, with an edge weight cut-off of 0.7 (for lipid metabolism) or 0.7 (for the PI signaling system), were visualized by Cytoscape. In the networks, the hub genes are those showing the most connections, and they often play important roles. In our study, genes with degree values between 100 and 150 were considered as mid-size hubs, and those with degree values >150 were considered as large hubs.

### Gene Annotation and Pathway Enrichment Analysis of WGCNA

Unigenes and pathways were annotated by searches against BLAST databases, including the Kyoto Encyclopedia of Genes and Genomes database (KEGG), Clusters of Orthologous Groups of proteins (COG), NCBI non-redundant protein sequences (Nr), and Swissprot. In addition, Gene Ontology (GO) analyses and KEGG enrichment analyses were conducted to detect potential functions of co-clustered genes in the conchosporangia maturation-related module. Pathways with *P* value < 0.05 were considered as significantly enriched.

### Cloning and Sequence Analysis of *P. haitanensis* DGK (*PhDGK1*)

We searched the unigene database of *P. haitanensis* for sequences (Unigene0032086) homologous to the conserved domains of *PhDGK1*. Head-to-toe primers (Supplementary Table 1) were designed and used to amplify the full-length *PhDGK1* cDNA by polymerase chain reaction (PCR). All procedures were performed according to the manufacturer’s protocol, as described by Chen et al. (2015). The sequence analysis of *PhDGK1* was performed according to Xiao et al. (2014).

### Gene Expression Analysis by qRT-PCR

The RNA used for quantitative reverse-transcription PCR (qRT-PCR) was the same as that for transcriptome sequencing. For the first-strand cDNA synthesis, the PrimeScript^TM^ RT Reagent Kit with the gDNA Eraser Kit (Takara, Kyoto, Japan) were used following the manufacturer’s instructions. The transcript levels of selected genes were verified by qRT-PCR with the following cycling conditions: 95°C for 30 s, followed by 40 cycles of 95°C for 10 s, 55°C for 10 s and 72°C for 20 s. The qRT-PCRs were conducted using SYBR^®^ Premix Ex Taq^TM^ II (Tli RNaseH Plus, Takara, Kyoto, Japan). The reactions were performed in 20 μL reaction volumes containing 10 μL of 2 × SYBR^®^ Premix Ex Taq, 0.8 μL each primer (10 μM concentration of each primer), 3.0 μL diluted cDNA mix, and 6.4 μL RNA-free water. A melting curve analysis of each amplicon was conducted to verify the specificity of the amplification reaction. No-template controls were included for each primer pair and each PCR reaction was carried out with three biological replicates. The *UBC* gene encoding ubiquitin-conjugating enzyme was used as the internal control (Li et al., 2014). The 2^–△^^△^^*Ct*^ method was used to calculate relative gene expression values (Livak and Schmittgen, 2001). The sequences of the primers used are listed in Supplementary Table 2. The concentration of all primers was 10 μM.

## Results

### Difference in Conchosporangia Maturity Between Strains S-1 and S-2

**FIGURE 1:**
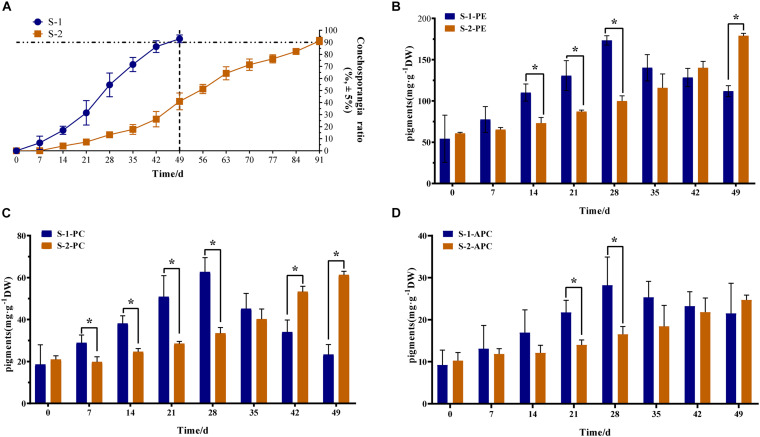
Phenotypic and phycobiliprotein content differences between S-1 and S-2 strains of *Pyropia haitanensis*. **(A)** Ratio of conchosporangia in S-1 and S-2 strains (*n* = 10). **(B)** Phycoerythrin (PE) content in S-1 and S-2 strain (n = 3). **(C)** Phycocyanin (PC) content in S-1 and S-2 strains (*n* = 3). **(D)** Allophycocyanin (APC) content in S-1 and S-2 strains (*n* = 3). Data are means ± SD. Statistical significance was calculated by Student’s *t*-test. Significant differences level: *P < 0.05.

The maturation of S-1 and S-2 strains showed obvious differences. The S-1 strain took 49 days to form conchosporangia, while the S-2 strain took 91 days (Figure 1A). The contents of phycobiliproteins (PE, PC, and APC) in S-1 and S-2 strains were determined (Figures 1B–D). From 7 days of maturation, the pigment contents differed significantly between the two strains. Additionally, the materials at time 0 day are the vegetative conchocelis and without conchosporangia, while that at the time 1 day are the earliest stages of conchosporangia maturation. Thus, we selected 0, 1, 7, and 49 days for the S-1 strain and 0, 1, 7, 49, and 91 days for the S-2 strain as time points for further analyses.

### Transcriptome Profiling

**FIGURE 2:**
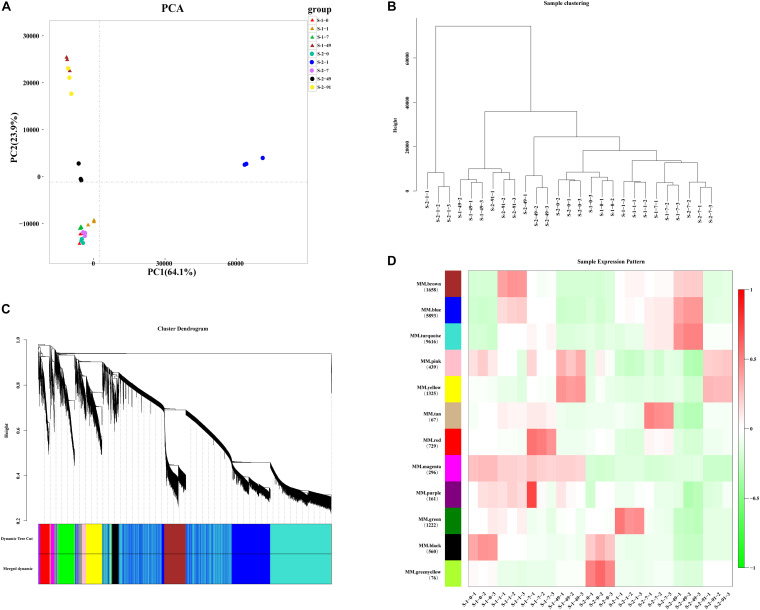
Principal coordinate analysis (PCA) and Weighted Gene Co-Expression Network Analysis (WGCNA) of *Pyropia haitanensis* transcript profiles. **(A)** PCA of expression patterns of 47,046 unigenes at different developmental stages in S-1 and S-2 strains. **(B)** Sample clustering of 27 samples, each smallest branch represents a sample, and each larger branch represents their clustering relationship. **(C)** Clustering dendrogram and modules for 22,042 unigenes. Each gene is represented by a leaf in the tree. Y axis represents network distance, as determined by topological overlap. Different colors show module membership after being merged. **(D)** Module–trait relationships. Colors on the left represent 12 modules and the numbers of unigenes are noted in each module. Heat-map shows the module eigengene correlations with traits (27 samples in this experiment).

After assembly and filtering, all clean reads were assembled into 47,046 unigenes with an average length of 926 bp, and average GC content of 63.65% (Supplementary Table 3). N50 that is defined as the sequence length of the shortest contig/Unigene at 50% of the total genome length is 1,489 bp. 27 cDNA libraries were generated by RNA sequencing. To assess the correlations among different libraries, a principal component analysis (PCA) was performed for three replicates per treatment (Figure 2A). The PCA analysis showed that the transcriptomic data were similar among the three replicates, while the expression patterns similarity among different treatments. S-1-49 and S-2-91 has the similar expression patterns, and S-1-0, S-1-1, S-1-7, and S-2-0, S-2-7 has the similar expression patterns, S-2-1 and S-2-49 has a special expression pattern. In the BLAST analyses, 19,331 (41.09%) unigenes were annotated with information from at least one of the following databases: the KEGG and COG, Nr and Swissprot, and 8,983 unigenes were annotated with information from all of these databases (Supplementary Table 4). To validate the quality of RNA-Seq data, 15 unigenes were selected for qRT-PCR analysis. The majority of them (75%) had a correlation coefficient ≥ 0.8 between RNA-Seq data and qRT-PCR results, indicating strong consistency between quantitative results and the transcriptome data (Supplementary Figure 1 and Supplementary Table 2).

### Modules Associated With Conchosporangia Maturation in WGCNA

In total, 25,004 unigenes were removed during filtering. A TOM was generated using a set of 22,042 unigenes for WGCNA (Figures 2B,C and Supplementary Tables 5, 6). The clustering results of WGCNA were similar to those of PCA, in that replicates of samples clustered together (Figure 2B). After a dynamic tree cut and merging, 12 modules were identified and differentiated using different colors, with gene numbers ranging from 67 (tan module) to 9,616 (turquoise module) (Figure 2D). Module–trait relationships and eigengenes expressions of each module indicated that the genes in the yellow module were up-regulated at the conchosporangia stage (Supplementary Figure 2A). The genes in the turquoise module were up-regulated during conchosporangia formation, and the turquoise module was characterized by two clear fluctuations (Supplementary Figure 2B). These fluctuations seemed to provide a clue as to the differences in conchosporangia maturity between the two strains, so the turquoise module was chosen for further analysis.

### Pathway Enrichment Analysis of Yellow Module

**FIGURE 3:**
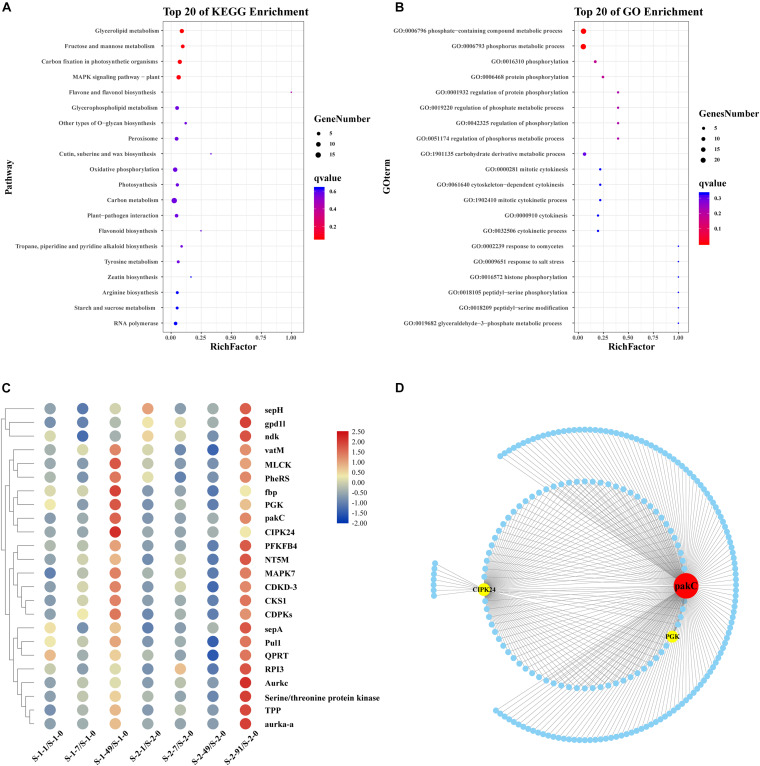
Functional enrichment analysis and network construction of yellow module obtained by WGCNA. **(A)** Top 20 KEGG pathways enriched with unigenes in yellow module. Enriched pathways with *P* < 0.05 are marked with a red dot. **(B)** Gene Ontology (GO) categories enriched with genes in yellow module. Red dots indicate those pathways with *P* < 0.05. **(C)** Hierarchical clustering of genes related to phosphorus metabolism in yellow module. Red: upregulation; Blue: downregulation. Transcript levels of genes are shown as -fold changes in RPKM compared with that at S-1-0 or S-2-0. sepH, cytokinesis protein; gpd1l, glycerol-3-phosphate dehydrogenase; ndk, nucleoside diphosphate kinase; vatM, vacuolar proton ATPase; MLCK, myosin light chain kinase; PheRS, phenylalanyl-tRNA synthetase; fbp, inositol phosphatase/fructose-16-bisphosphatase; PGK, phosphoglycerate kinase; pakC, p21-activated protein kinase; CIPK24, CBL-interacting protein kinase 24; PFKFB4, 6-phosphofructo-2-kinase/fructose-2, 6-biphosphatase 4; NT5M, 5 (3)-deoxyribonucleotidase; MAPK7, mitogen-activated protein kinase 7; CDKD-3, cyclin-dependent kinase D-3; CKS1, cyclin-dependent kinase; CDPKs, calcium-dependent protein kinase; sepA. serine protease autotransporter; Pull, pullulanase; QPRT, nicotinate-nucleotide pyrophosphorylase; RPI3, ribose-5-phosphate isomerase; Aurkc, aurora kinase C; Tpp, tyrosine-protein phosphatase; aurka-a, aurora kinase. **(D)** Sub-network for hub genes in phosphorus metabolism and candidate hub-interacting genes. Sub-network was built by extracting hub genes and candidate co-expressed genes were those connected to more than one hub gene in phosphorus metabolism. pakC, p21-activated protein kinase; CIPK24, CBL-interacting protein kinase 24; PGK, phosphoglycerate kinase.

The yellow module contained 1,325 unigenes. The functional pathways were characterized using GO and KEGG enrichment analyses. The KEGG pathway enrichment analysis (*P* < 0.05) revealed that many unigenes in the yellow module were involved in metabolism and genetic information, while many in the turquoise module were involved in glycerolipid metabolism, fructose and mannose metabolism, carbon fixation in photosynthetic organisms, and MAPK signaling pathway-plant (Figure 3A). The GO analysis of unigenes in the yellow module grouped many unigenes in the “Biological Process” and “Molecular Function” categories. The subcategories most enriched with unigenes were catalytic activity, metabolic process, and cellular process (Figure 3B). In the “Biological Process” category, 24 unigenes were grouped in the phosphorus metabolic process subcategory (Figure 3B). The heatmap cluster analysis identified highly expressed genes related to phosphorus metabolism at the conchosporangia stage (Figure 3C). The co-expression network for phosphorus metabolic process based on the yellow module contained 211 nodes and 294 edges (Figure 3D and Supplementary Data 1). Two phosphorus metabolic process genes exhibiting high connectivity (>100) were considered as large hubs (indicated in red in Figure 3D). These large hubs were annotated as p21-activated protein kinase (unigene0002810/pakC) and CBL-interacting protein kinase 24 (unigene0029817/CIPK24).

### Pathway Enrichment Analysis of Turquoise Module

**FIGURE 4:**
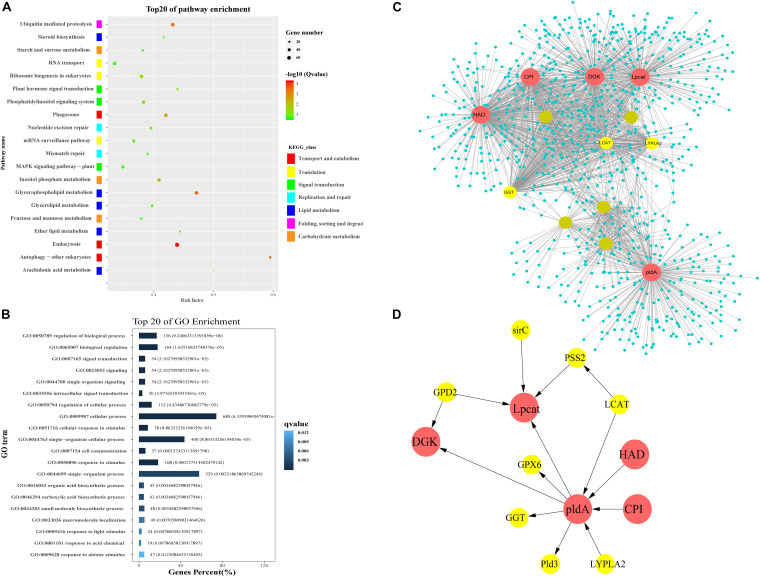
Functional enrichment analysis and network construction of turquoise module. **(A)** Top 20 KEGG pathways enriched with genes in turquoise module. **(B)** Top 20 GO subcategories enriched with genes in turquoise module. **(C)** Network for lipid metabolism in turquoise module. Network was constructed by extracting turquoise genes annotated with lipid metabolism as seed nodes, with an edge weight cut-off of 0.7. Red dots indicate hub genes in the network; yellow dots indicate nodes with connectivity of at least 50. Node size represents level of connectivity. **(D)** Co-expression network for hub genes in lipid metabolism and candidate hub-interacting genes. Sub-network was built by extracting hub genes and the selected candidate co-expressed genes connected to more than one hub genes within the lipid metabolism network. HAD, haloacid dehalogenase; DGK, diacylglycerol kinase; pldA, phospholipase D; Lpcat, lysophospholipid acyltransferase; CPI, cyclopropyl isomerase; LCAT, lecithin-cholesterol acyltransferase; GGT, gamma-glutamyl transferase; LYPLA2, lysophospholipase 2; PSS2, CDP-diacylglycerol-serine *O*-phosphatidyltransferase; GPD2, glycerol-3-phosphate dehydrogenase; GPX, glutathione peroxidase; Pld3, phospholipase d y-like protein; sirC, cytokinin hydroxylase-like.

The turquoise module contained 9,616 unigenes. Many unigenes were involved in genetic and environmental information processing and metabolism. The GO analysis of unigenes in the turquoise module indicated that many of them were grouped into the “Biological Process” and “Molecular Function” categories. In the “Molecular Function” category, the subcategories most enriched with unigenes in the turquoise module were binding and catalytic activity (Figure 4B). The KEGG pathway enrichment analysis (*P* < 0.05) revealed that unigenes in the turquoise module were related to endocytosis, glycerophospholipid metabolism, ubiquitin mediated proteolysis, inositol phosphate metabolism, and the phosphatidylinositol (PI) signaling system (Figure 4A). As indicated by the KEGG classes, glycerophospholipid metabolism, arachidonic acid metabolism, ether lipid metabolism, glycerolipid metabolism, and steroid biosynthesis are components of lipid metabolism. We subsequently constructed co-expression networks to screen for hub genes in lipid metabolism and the PI signaling system.

### Identification of Key Genes Related to Lipid Metabolism

A lipid metabolism network was built using genes related to lipid metabolism (glycerophospholipid metabolism, arachidonic acid metabolism, ether lipid metabolism, glycerolipid metabolism, and steroid biosynthesis) and their co-expressed genes in the turquoise module (Supplementary Data 2). The network contained 1,193 nodes and 3,097 edges. Five lipid metabolism-related genes exhibiting high connectivity (>200) were identified as large hubs (indicated in red in Figure 4C). These large hub genes were annotated as haloacid dehalogenase superfamily protein (unigene0005058/HAD), diacylglycerol kinase (unigene0032086/DGK), phospholipase D (unigene0019891/pldA), lysophospholipid acyltransferase (unigene0036610/Lpcat), and cyclopropyl isomerase (unigene0019326/CPI). There were three mid-size hub genes (connectivity > 100), which were annotated as lecithin-cholesterol acyltransferase (unigene0002518/LCAT), gamma-glutamyl transferase (unigene0022019/GGT), and lysophospholipase 2 (unigene0015050/CPLA2) (Figure 4D).

### Identification of Key Genes Related to PI Signaling System

**FIGURE 5:**
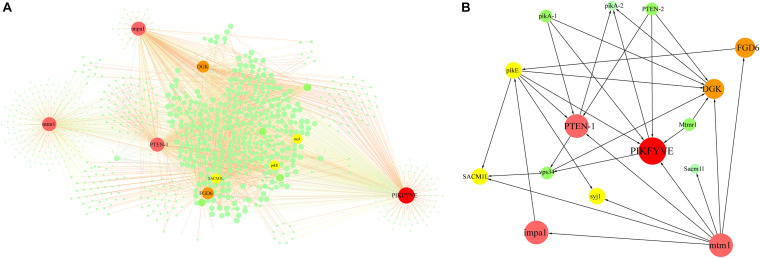
Network construction related to phosphatidylinositol signaling system enriched from turquoise module. **(A)** Network for PI signal system in turquoise module. Network was constructed by extracting turquoise module genes with annotations related to PI signaling system as seed nodes, with an edge weight cut-off of 0.7. Red dots indicate hub genes in the network, yellow dots indicate nodes with connectivity of at least 100. Node size represents level of connectivity. **(B)** Co-expression network for hub genes in PI signal system and candidate hub-interacting genes. Sub-network was built by extracting hub genes and selected candidate co-expressed genes connected to more than one hub gene within the PI signal system network. PIKFYVE, 1-phosphatidylinositol-3-phosphate 5-kinase; PTEN-1, phosphatidylinositol-3, 4, 5-triphosphate 3-phosphatase; mtm1, phosphatases II; impa1, inositol monophosphatase 3; DGK, Diacylglycerol kinase; FGD6, pleckstrin domain-containing protein; SACM1L, phosphoinositide phosphatase SAC7; pikE, phosphatidylinositol 3-kinase; syj1, inp53-like protein; Mtmr1, myotubularin-related protein 1; PTEN-2, phosphatidylinositol-3, 4, 5-triphosphate 3-phosphatase; pikA-1, Phosphatidylinositol 3-kinase; vps34, phosphatidylinositol 3-kinase.

In the turquoise module-based co-expression network for the PI signal system, the PI signal network contained 1,028 nodes and 3,113 edges (Figure 5A and Supplementary Data 3). Six PI signal system-related genes exhibiting high connectivity (>250) were identified as large hubs (indicated in red in Figure 5B). These large-hub genes were annotated as 1-phosphatidylinosito-l-3-phosphate-5-kinase (unigene0017302/PIKFYVE), phosphatidylinositol-3,4,5-trisphosphate-3-phosphatase and dual-specificity protein phosphatase (unigene0018660/PTEN-1), phosphatase II (unigene0004956/mtm1), inositol monophosphatase 3 (unigene0005716/impa1), diacylglycerol kinase (unigene0032086/DGK), and pleckstrin domain-containing protein (unigene0009349/FGD6). Among these hubs, PIKFYVE had the highest connectivity. PTEN-1, mtm1, and impa1 not only participated in the phosphatidylinositol signaling system, but also in inositol phosphate metabolism.

### Cloning, Sequence, and qPCR Analysis of *P. haitanensis* DGK (*PhDGK1*) Genes

**FIGURE 6:**
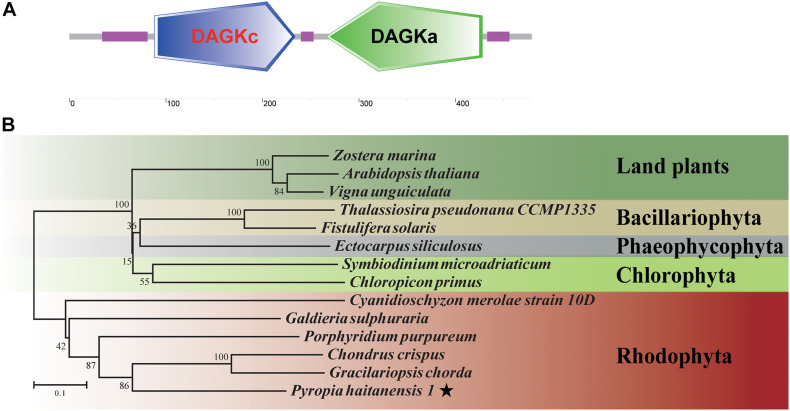
Analysis of conserved domains and phylogenetic relationships of *PhDGK1*. **(A)** Protein-conserved domain prediction of *PhDGK1*. Green box indicates diacylglycerol kinase accessory domain (DGKa), blue box indicates diacylglycerol kinase catalytic domain (DGKc), purple boxes indicate regions of low complexity. **(B)** Phylogenetic relationships of *PhDGK1* from *P. haitanensis* and related proteins from representative Cyanophyta, Chlorophyta, Phaeophyta, and land plant species. Phylogenetic tree was constructed using the maximum-likelihood method. Bootstrap values after maximum-likelihood analysis and posterior probabilities after the Bayesian analysis are indicated at nodes and branches, respectively.

**FIGURE 7:**
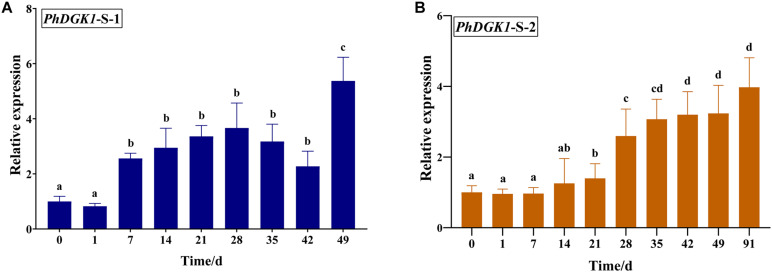
Relative transcript levels of *PhDGK1* at different time points during conchosporangia maturation in *P. haitanensis* S-1 strain **(A)** and S-2 strain **(B)**. Different letters indicate significant difference (*P* < 0.05).

Based on the sequence of unigene0032086, encoding a diacylglycerol kinase, the full-length cDNA of *PhDGK1* was amplified using two head-to-toe primers (PhDGK1F and PhDGK1R). The nucleotide sequence of *PhDGK1* was 1,440 bp long (Supplementary Table 7). Domain searches showed that amino acids 102–248 comprise the diacylglycerol kinase catalytic domain (DGKc), and amino acids 280–442 comprise the diacylglycerol kinase accessory domain (DGKa) (Figure 6A). A domain analysis indicated that PhDGK1 belongs to DGK Cluster II. A phylogenetic tree supported the existence of a sister-group relationship between *P. haitanensis* and other Rhodophyta species, but implied that Pyropia species diverged from Cyanophyta, Chlorophyta, Phaeophyta, and land plants (Figure 6B). We determined the dynamic changes in the transcript levels of *PhDGK1* in the S-1 strain and S-2 strain: *PhDGK1* transcript levels increased significantly on 7 days of maturation and remained high in the S-1 strain, but increased significantly on 14 days of maturation in the S-2 strain (Figure 7).

### Detection of Genes and Pathways Interaction With PA

**FIGURE 8:**
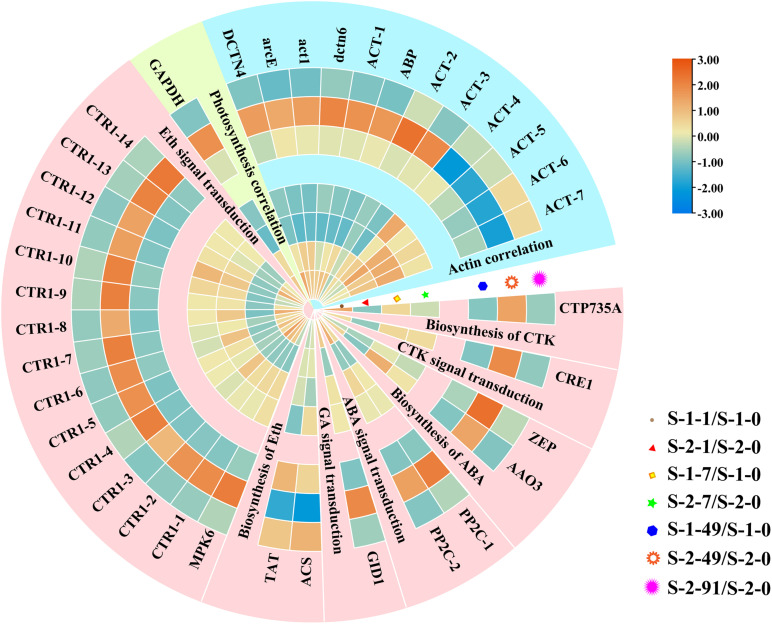
Hierarchical clustering of downstream genes coordinated by PA. Red: up-regulation; Blue: down-regulation. Cerulean style indicates genes related to photosynthesis, pale yellow style indicates genes related to and actin rose red and style indicates genes related to plant hormone synthesis and signal transduction. Transcript levels of genes are shown as -fold changes in RPKM compared with that in S-1-0 or S-2-0. DCTN4, Dynactin p62; arcE, actin related protein 2/3; act1, actin type I; dctn6, Dynactin subunit 6; ACT, actin; ABP, actin binding protein; GAPDH, glyceraldehyde 3-phosphate dehydrogenase; CTP735A, cytokinin hydroxylase; CRE1, Sensory transduction histidine kinase; ZEP, Zeaxanthin epoxidase; AAO3, indole-3-acetaldehyde oxidase; PP2C, protein phosphatase 2C; GID1, hormone-sensitive lipase; ACS, 1-aminocyclopropane-1-carboxylate synthase; TAT, tyrosine aminotransferase; MPK6, Mitogen-activated protein kinase; CTR, constitutive triple response 1.

Analyses of gene expression data in the turquoise module of WGCNA revealed that the key pathways and genes that interacted with PA were photosynthesis, plant hormone synthesis and signal transduction, and actin protein (Figure 8). These genes tended to be expressed at high levels at the mature conchosporangia stage, and at higher levels in the S-1 strain than in the S-2 strain. As shown in the figure, except for the constitutive triple response 1-10 (*CTR1-10*) gene, the other *CTR1* genes were expressed at higher levels in the S-1 strain than in the S-2 strain.

## Discussion

In this study, we focused on two strains with differences in conchosporangia maturation. The pigment content differed significantly between the S-1 and S-2 strains from the 7th day of maturation onwards (Figure 1). In particular, the ratio of conchosporangia in the S-1 strain was more than 7% on day 7, while the S-2 strain had not yet formed conchosporangia at this time point (Figure 1A). Moreover, the complete maturation period was longer in the S-2 strain than in the S-1 strain. These differences suggest that there may be differences in response mechanisms between the S-1 and S-2 strains. Genetic transformation methods are not well developed for seaweeds, so mutants were not available to study the mechanisms of conchosporangia maturation. Instead, these two strains were selected as favorable materials for studying the molecular mechanisms of the maturation of conchosporangia in *P. haitanensis*.

We identified the expression of genes in different modules at different time points during the maturation period using RNA-seq and WGCNA analyses. Our data indicate that phosphorus metabolic processes, lipid metabolism, and the PI signaling system are important metabolic pathways during conchosporangia maturation in *P. haitanensis*. The development and maturation of plants depends on regulation of signaling systems. The PI signaling system, which includes a series of kinases and phosphatases, is involved in the perception and transduction of external stimulation. In this study, the PI signaling pathway showed significant enrichment in the conchosporangia maturity-related module, supporting its crucial function in conchosporangia maturation in *P. haitanensis*. Phosphatidylinositol-3-phosphate-5-kinase (PIKFYVE) is required for the production of phosphatidylinositol-3,5-bisphosphate [PI(3,5)P2], a positive regulator in the PI signaling system, and plays roles in nitrogen-regulated mitotic commitment and cell size control (Cobley et al., 2017). The [PI(3,5)P2] synthesized by proteins in the PIKFYVE family plays roles in modulating the dynamics of vacuolar rearrangement, which is essential for successful pollen development in *Arabidopsis* (Whitley et al., 2009). Proteins in the PIKFYVE family have also been implicated in endomembrane trafficking and in the regulation of membrane recycling, vacuolar pH, and in the homeostatic control of reactive oxygen species during pollen tube growth (Serrazina et al., 2014). In this study, *PIKFYVE* showed different expression patterns between the S-1 and S-2 strains (Supplementary Figure 3). The upregulation of *PIKFYVE* occurred earlier in the S-1 strain than in the S-2 strain, indicating that PIKFYVE might promote earlier maturation in the S-1 strain. In addition to mediating the PI signal pathway, PIKFYVE also functions in the development of microtubules (Hirano et al., 2015). In this study, the increase in *PIKFYVE* expression was accompanied by an increase in the expression of microtubule-associated genes (Supplementary Figure 4). This result suggests that PIKFYVE might promote conchosporangia maturation in *P. haitanensis* via not only regulating PI signaling system but also mediating cell size.

Additionally, DGK is a key factor of PI turnover and initiates PI regeneration (Sakane et al., 2017). In Arabidopsis, DGKs are encoded by seven genes (*AtDGK1* to *AtDGK7*) grouped into three clusters (clusters I, II, and III) (Gomez-Merino et al., 2005). *AtDGK2* and *AtDGK4* belong to DGK Cluster II, and encode ER-localized DGKs that are required for vegetative and gametophyte development as well as glycerolipid metabolism (Angkawijaya et al., 2020). *AtDgk4*-knockout mutants were found to be defective in pollen tube growth (Vaz Dias et al., 2019). Here, DGK as the core gene was enriched in both the PI pathway and the lipid module (Figures 4, 5). Therefore, we obtained the full-length *PhDGK1* through direct PCR. *PhDGK1* comprises DGKc and DGKa; the former combines with PIP2 hydrolysis to generate a PA from DGK/PLC during the signal transduction cascade in eukaryotic cells (Kue Foka et al., 2020). Our sequence analyses indicated that *PhDGK1* does not contain two C1-type domains or a calmodulin-binding domain. These features indicate that *PhDGK1* belongs to DGK Cluster II. Thus, PhDGK1 is the same type of enzyme as DGK2 and DGK7, which are known to be involved in plant growth and development. For example, *MpDGK2* from *Malus prunifolia* was shown to affect the growth and tolerance of *Arabidopsis via* regulating H_2_O_2_ homeostasis and antioxidant enzyme activity (Tang et al., 2020). In addition, the content of PA, an important signaling molecule, was decreased in an *AtDGK2-*knockdown mutant, resulting in deceased pollen viability and pollen germination (Angkawijaya et al., 2020). In this study, both the RNA-seq and qRT-PCR data showed that *PhDGK1* was up-regulated in the S-1 and S-2 strains during conchosporangia maturation (Supplementary Figures 2, 3). Moreover, *PhDGK1* was significantly upregulated earlier in the S-1 strain than in the S-2 strain, and its transcript levels were always higher in the S-1 strain than in the S-2 strain (Figure 7). Accordingly, *PhDGK1* might be as the candidate hub gene to promote the maturation of conchosporangia.

A previous study showed that PLC functions upstream of DGK and participates in the synthesis of DGK (Kue Foka et al., 2020). In the current study, we detected similar expression trends of *PLC* and *DGK* (Supplementary Figure 4). PA is mainly produced from major membrane phospholipids and diacylglycerol (DAG) by DGK and PLC (Arisz et al., 2009). PA is a precursor for the biosynthesis of most classes of glycerolipids, which are the major components of cellular membranes. Glycerolipids play important roles in plant growth and development by coordinating the expression of downstream genes (Munnik, 2001; Wang et al., 2016). Downstream proteins that bind to PA include proteins related to cell walls, hormone synthesis and signal transduction, and photosynthesis (Yao and Xue, 2018). For example, PA can directly bind to phosphoenolpyruvate carboxylase (PEPC) isoforms and then regulate photosynthesis by affecting PEPC activity (Monreal et al., 2010). Zhang et al. (2012) found that PA could bind to microtubule-associated protein 65-1 (MAP65-1) to stimulate its interaction with microtubules, hence increasing the polymerization of cortical microtubules. In addition to microtubules, PA levels are important for actin cytoskeleton dynamics through their role in regulating actin-capping proteins (CPs) (Yao and Xue, 2018). The binding of CPs with PA reduces their activity, which promotes the reorganization of actin as plants adapt to various environmental conditions (Galvan-Ampudia et al., 2013). Mishra et al. (2006) showed that PAs mediate ABA-stimulated stomatal opening/closure through interacting with phosphatase 2C (PP2C) and the Gα subunit of G protein. Binding of PA with the CTR1 protein in the ethylene signal transduction pathway results in inhibition of its kinase activity and disrupts its intra and intermolecular interactions, thereby promoting the ethylene response (Testerink et al., 2007). Here, the cluster analysis of the genes in the turquoise module showed that genes involved in actin dynamics, phytohormone synthesis, and signal transduction (PP2C and CTR1) were up-regulated during conchosporangia maturation (Figure 8). The transcript levels of these genes were higher in the S-1 strain than in the S-2 strain. During maturation of *Pyropia* conchosporangia, the conchocelis cell wall and cell membranes undergo significant changes (Zhong et al., 2016). The results of the present study suggest that the crosstalk among PA, actin, and phytohormones might be conducive to the rapid transformation of the conchocelis cell wall and the maturation of conchosporangia.

Furthermore, *PTEN*, *Lpcat*, and other genes involved in PI signal transduction and lipid metabolism were also enriched in co-expression work (Figures 4C,D, 5B), supporting their roles in the process of conchocelis maturation in *P. haitanensis*. The degradation of PtdIns(3)P was almost lost in the *PTEN*-deficient cells treated with an inhibitor of PIKFYVE, suggesting that *PIKFYVE* and *PTEN* are two major players in PtdIns(3)P metabolism (Hazeki et al., 2012). Yang et al. (2019) also found that DGK, Lpcat and PLD participate in the oil accumulation in developing Soybean Seeds by WGCNA analyze. However, the interactions between these hub genes need further validated.

## Conclusion

We screened two strains of *P. haitanensis* with obvious differences in conchocelis maturation. Further analyses revealed that lipid metabolism is an important metabolic pathway in the process of conchosporangia maturation. Several hub genes encoding DGK and PIKFYVE were identified by WGCNA. Our results indicate that *PhDGK1* not only participates in the formation of conchosporangia, but also affects the speed of conchosporangia maturation. Additionally, PA is likely to be involved in conchosporangia maturation by mediating actin interactions and phytohormone signal transduction. The data presented here provide new insights into the conchocelis development of *Pyropia*/*Porphyra*.

## Data Availability Statement

The datasets presented in this study can be found in online repositories. The names of the repository/repositories and accession number(s) can be found below: https://www.ncbi.nlm.nih.gov/genbank/, MW687621 and https://www.ncbi.nlm.nih.gov/, PRJNA681100.

## Author Contributions

CX, WW, and YL conceived and designed the experiment. YL and WW performed the experiments and data analysis. CX and CC contributed by planning, supervising, and financing the work. DJ, KX, and YX helped to prepare the materials and reagents. YL and WW drafted and revised the manuscript. All authors read and approved the final manuscript.

## Conflict of Interest

The authors declare that the research was conducted in the absence of any commercial or financial relationships that could be construed as a potential conflict of interest.

## Supplementary Material

Supplementary Figure 1 Relative transcript levels of selected genes of *Pyropia haitanensis* at different times during conchosporangia maturation as determined by qRT-PCR.

Supplementary Figure 2 Eigengene expression profiles for yellow **(A)** and turquoise **(B)** modules. Bar-plot shows eigengene expression at each sampling point. For heat-maps, rows correspond to genes, columns correspond to samples, green denotes under-expressed genes, red denotes over-expressed genes.

Supplementary Figure 3 Hierarchical clustering of PI signal system. Red: up-regulation; Blue: down-regulation. Transcript levels of genes are shown as fold changes of RPKM compared with that at S-1-0 or S-2-0.

Supplementary Figure 4 Hierarchical clustering of genes related to microtubules **(A)** and PLC **(B)**. Red: up-regulation; Blue: down-regulation. Transcript levels of genes are shown as -fold changes of RPKM compared with that at S-1-0 or S-2-0.

Supplementary Table 1 Gene-specific primers for cloning *PhDGK1* and quantifying its expression.

Supplementary Table 2 Gene-specific qRT-PCR primers for verifying differentially expressed genes in *Pyropia haitanensis*.

Supplementary Table 3 Overview of *Pyropia haitanensis* transcriptome information.

Supplementary Table 4 Overview of *Pyropia haitanensis* annotation information.

Supplementary Table 5 All list and information of WGCNA. All.kTotal: Connectivity of all edges of the gene. All.kWithin: The degree of connectivity under this module, the sum of correlation coefficients between a gene and other genes. MM.XXX: The correlation coefficient between the gene and the eigenvalues of XXX module. MM.XXX.pvalue: The *P* value of the MM value of the gene and the characteristic value of XXX module. XXX meaning of the different colors of Modules.

Supplementary Table 6 The annotation of gene in different Module. Symbol: The symbol number of gene. Description: annotation of Nr database. KEGG_A_class: KEGG first Level Notes. KEGG_B_class: KEGG second Level Notes. Pathway: annotation of KEGG pathway. GO Component: annotation of cell component. GO Function: annotation of GO molecular function. GO Process: annotation of GO biological process.

Supplementary Table 7 The nucleotide and amino acid sequence information of *PhDGK1*. Red represented the initiator and terminator codons, green represented the primers located.

## Abbreviations

APC: allophycocyanin
*CTR1*: constitutive triple response 1
DGK: diacylglycerol kinase
DGKa: diacylglycerol kinase accessory
DGKc: diacylglycerol kinase catalytic
GO: Gene Ontology
KEGG: Kyoto Encyclopedia of Genes and Genomes database
PA: phosphatidic acid
PC: phycocyanin
PCA: principal component analysis
PCR: polymerase chain reaction
PE: phycoerythrin
PLC: phospholipase C
PI: phosphatidylinositol
PIKFYVE: phosphatidylinositol-3-phosphate-5-kinase
qRT-PCR: quantitative reverse-transcription PCR
RPKM: reads per kilobase of transcript per million mapped reads
TOM: topological overlap matrix
WGCNA: weighted gene co-expression network analysis.
