# Supplementary figures and images for: Transcriptome Co-expression Network Analysis Identifies Key Genes Regulating Conchosporangia Maturation of Pyropia haitanensis
Source: Front Genet. 2021 Jun 30;12:680120. doi: 10.3389/fgene.2021.680120 (PMC8278576; doi:10.3389/fgene.2021.680120)

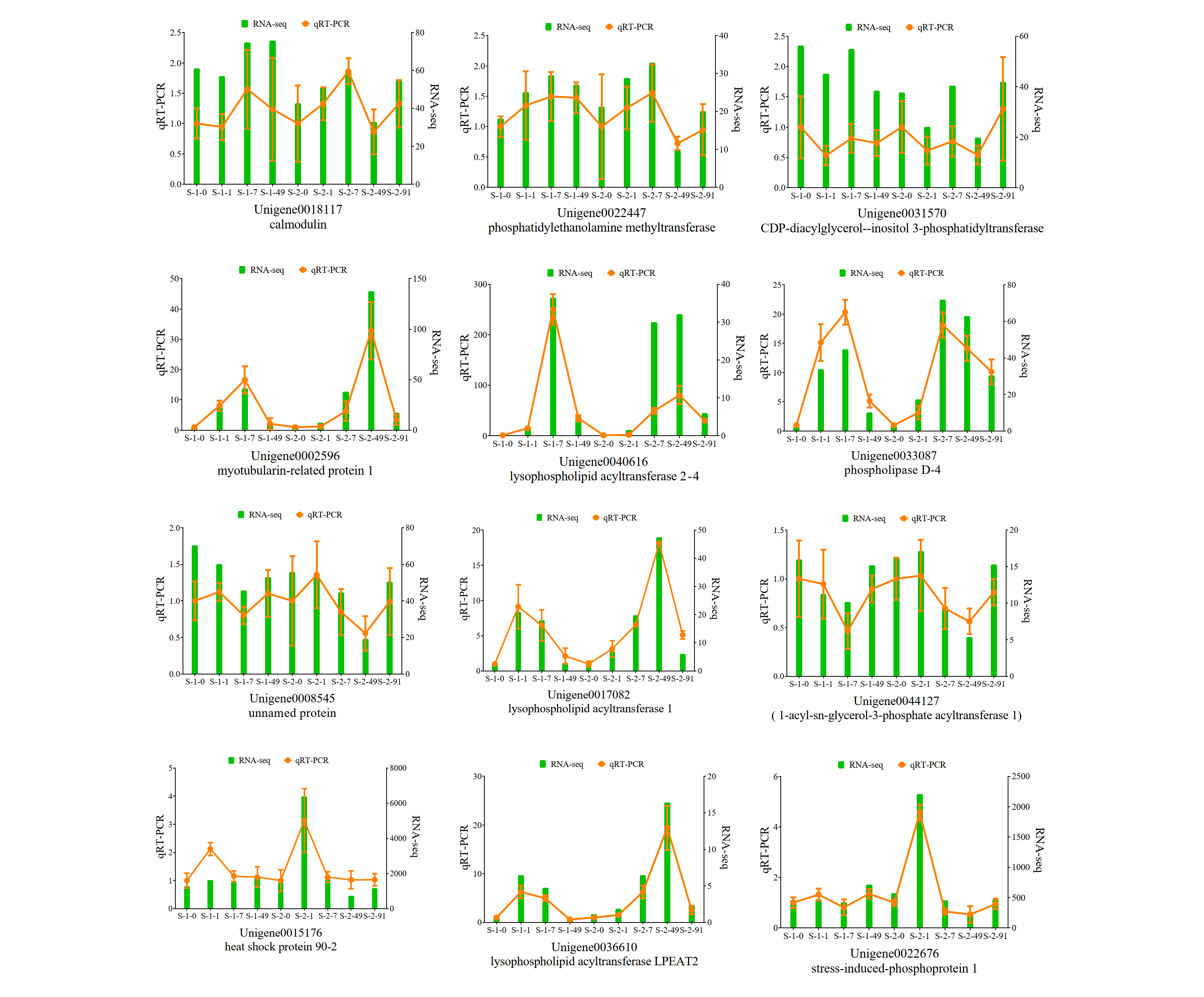

Supplement: Supplementary Figure 1 — Relative transcript levels of selected genes of Pyropia haitanensis at different times during conchosporangia maturation as determined by qRT-PCR. [file Image_1.TIF]

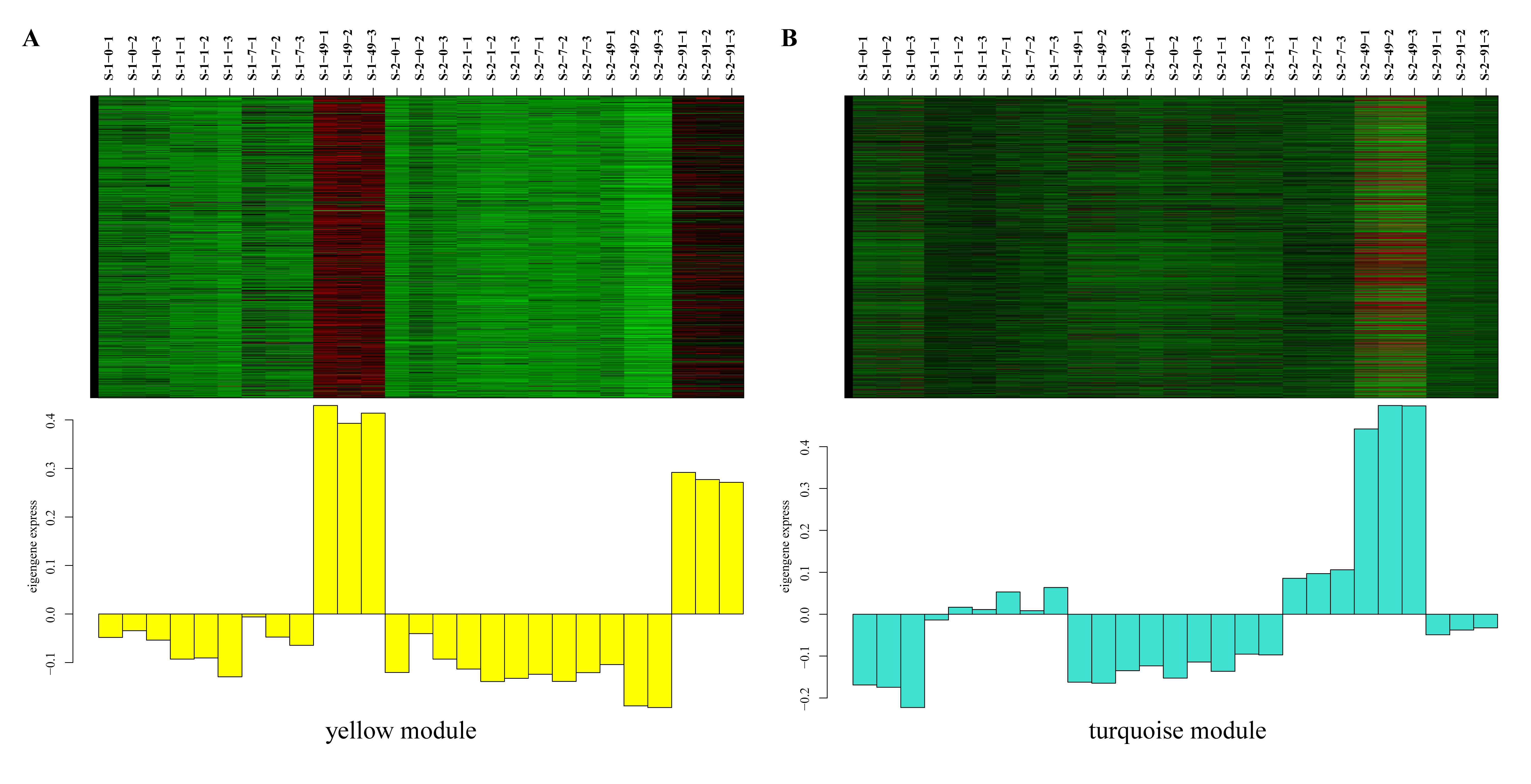

Supplement: Supplementary Figure 2 — Eigengene expression profiles for yellow (A) and turquoise (B) modules. Bar-plot shows eigengene expression at each sampling point. For heat-maps, rows correspond to genes, columns correspond to samples, green denotes under-expressed genes, red denotes over-expressed genes. [file Image_2.TIF]

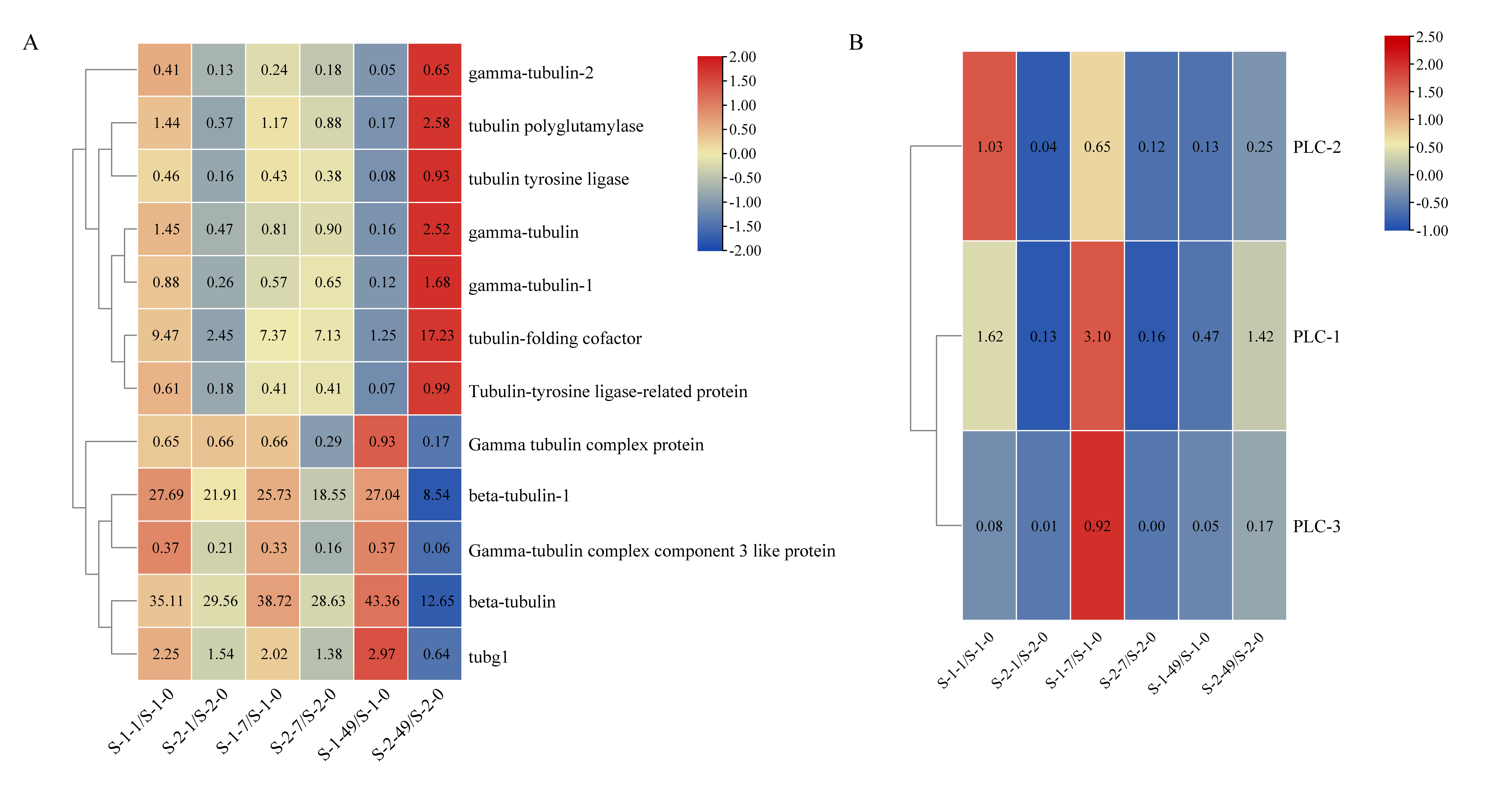

Supplement: Supplementary Figure 3 — Hierarchical clustering of PI signal system. Red: up-regulation; Blue: down-regulation. Transcript levels of genes are shown as fold changes of RPKM compared with that at S-1-0 or S-2-0. [file Image_3.TIF]
